# Countering Brutality to Wildlife, Relationism and Ethics: Conservation, Welfare and the ‘Ecoversity’

**DOI:** 10.3390/ani1010161

**Published:** 2011-01-27

**Authors:** Steve Garlick, Julie Matthews, Jennifer Carter

**Affiliations:** 1 Centre for Urban and Regional Studies, University of Newcastle, New South Wales, Australia; 2 Sustainability Research Centre, University of the Sunshine Coast, Queensland, Australia; E-Mail: jmatthew@usc.edu.au; 3 School of Social Sciences, University of the Sunshine Coast, Queensland, Australia; E-Mail: jcarter@usc.edu.au

**Keywords:** wildlife, ecoversity, Derrida, ethics, universities

## Abstract

**Simple Summary:**

Wildlife cruelty is commonplace in society. We argue for a new engagement with wildlife through three elements: a relational ethic based on intrinsic understanding of the way wildlife and humans might view each other; a geography of place and space, where there are implications for how we ascribe contextual meaning and practice in human-animal relations; and, engaged learning designed around our ethical relations with others, beyond the biophysical and novel, and towards the reflective metaphysical. We propose the ‘ecoversity’, as a scholarly and practical tool for focusing on the intersection of these three elements as an ethical place-based learning approach.

**Abstract:**

Wildlife objectification and cruelty are everyday aspects of Australian society that eschew values of human kindness, empathy, and an understanding of the uniqueness and importance of non-human life in the natural world. Fostered by institutional failure, greed and selfishness, and the worst aspects of human disregard, the objectification of animals has its roots in longstanding Western anthropocentric philosophical perspectives, post colonialism, and a global uptake of neoliberal capitalism. Conservation, animal rights and welfare movements have been unable to stem the ever-growing abuse of wildlife, while ‘greenwash’ language such as ‘resource use’, ‘management’, ‘pests’, ‘over-abundance’, ‘conservation hunting’ and ‘ecology’ coat this violence with a respectable public veneer. We propose an engaged learning approach to address the burgeoning culture of wildlife cruelty and objectification that comprises three elements: a relational ethic based on intrinsic understanding of the way wildlife and humans might view each other [1,2,3]; geography of place and space [4], where there are implications for how we ascribe contextual meaning and practice in human-animal relations; and, following [5], engaged learning designed around our ethical relations with others, beyond the biophysical and novel and towards the reflective metaphysical. We propose the ‘ecoversity’ [6], as a scholarly and practical tool for focusing on the intersection of these three elements as an ethical place-based learning approach to wildlife relationism. We believe it provides a mechanism to help bridge the gap between human and non-human animals, conservation and welfare, science and understanding, and between objectification and relationism as a means of addressing entrenched cruelty to wildlife.

## 1. Introduction

Everybody knows what a terrifying and intolerable picture a realist could paint of the physical, industrial, mechanical, chemical, hormonal, and genetic violence to which man has been submitting animal life for the past two centuries. Everybody knows what the production, breeding, transport, and slaughter of these animals has become [1].

In the early twenty-first century the brutal treatment of animals has reached a horrifying zenith. The burgeoning culture of animal cruelty is such that as Derrida [1,2,3] observes, everybody knows of the grim and horrifying pain and suffering to which we subject animals mass-produced for food and human consumption. But we allow ourselves to remain psychologically distant from this suffering. We may understand, but do not let the moral sensitivity of our humanness comprehend.

The welfare circumstance for production animals is horrific enough, but what hope is there for wildlife? What hope is there for animals whose wildness and purported freedom we cannot possess or harness, whose purpose we do not understand, whose language we cannot relate to, but whose finitude reminds us of our own mortality, failings and vulnerability on this planet? What hope is there for animals whose value and importance to us does not balance our neoliberal instrumental scales, and whose survival relies on our reorganizing our institutions, curbing our greed, and replacing anthropocentric disregard with respect, understanding, wonderment and recognition of the unique right of non-human beings and their offspring to life and a future?

To address these questions, this paper will proceed by first providing an account of animal cruelty and objectification which challenges the distinctions between animals and humans, and examines animal ethics from a geographical perspective to discuss ethical space and place. The three strands of this approach tease out a relational ethic informed by geography of place and space [4,7,8] and a Derridian [1,2,3] deconstruction of normative ways of addressing animal/human relations which challenge conventional ethics and its inability to truly represent the animal. The third strand of this approach is developed in the final section of this paper through an argument for the design of a structural approach to engaged learning, which goes beyond the biophysical and novel, to encompass ethical relations with animals within a reflective learning model. We tie these arguments to a practical and theoretical proposal for higher education innovation. The ‘ecoversity’ approach we propose [6], provides a means towards change which embeds an ethical approach to wildlife in everyday university practice and operations that will assist in bridging the gap between human and non-human animals, between conservation and welfare, between science and understanding, and between anthropocentrism and relationism in many areas that are a cause of cruelty to wildlife in diverse spaces today.

## 2. Animal Cruelty and Objectification

The dominant structures of capitalist economies frame nature-society relations in the context of commercialized agriculture, urbanization, industrialization, and a technological fix for all potentially limiting factors. These practices, in turn, sustain animal cruelty and objectification through disregard for otherness [7,8]. Perhaps one of the cruelest interactions between people and animals is demonstrated by industrial agriculture, where images of caged or confined animals (pigs, chickens and cows in particular) unable to move and suffering an inhumane existence are now commonplace. Animal objectification is exemplified by this horrific industry. Ransom [9], for example, argues that institutional isomorphism governs animal welfare standards to the extent that ‘the organizational form has the potential to take precedence over the content’, giving the welfare of animals (their presumed objective) the lowest priority. Even when attempting to promote the welfare of animals, structural forces frame and restrict our thinking about them to a prescriptive, scientistic, legalistic, technocratic, bureaucratic, indeed anthropocentric, framework. This makes it difficult, if not impossible, to consider the existence of animals in their own terms. Rather, their existence is instrumental to serving human comfort and desire. A relational ethics approach is needed to complement the scientistic technology paradigm that dominates welfare and conservation discourse as it relates to wildlife and farm production animals.

While consumers in some parts of the world are increasingly calling for foods produced only with ethical treatment of animals, [8] the eco-friendly branding of such commodified animals usually demands a higher price and relies on anthropocentric forms of accreditation. Increasingly, with global financial and climate crises, the lower and middle class majority will be unable to afford these more humanely-produced alternatives and the dominant structures of power and money will necessitate further animal commodification for utilitarian purposes at economies of scale that only intensify cruelty and objectification.

In the law, animal welfare is mostly considered from a property perspective. Wildlife welfare and protection from cruelty under the law are more challenging, bound up in a range of anthropocentric jurisdictional legislation and bureaucratic convenience underpinned by poor data, analysis and inference, and industry vested interests—all of which further objectify the animal.

Such legalistic, bureaucratic and technocratic paradigms dominate public thinking about the human-nature connection and are similarly applied to the treatment of native animals. In planning frameworks, for example, animal subjectivities are rarely acknowledged, instead being replaced by generally superficial analysis of numbers, leading to statistically and scientifically vague concepts such as ‘endangered’, ‘threatened’, ‘abundant’. As Wolch [7] explains, planning regulations are anthropocentrically driven, introduced by local government authorities that continue to legitimize urban expansion (housing, commercial development, transport, entertainment) into natural areas, using narrow interpretations of ecology and economy that suit their own purposes. ‘Green’ developments supposedly resolve the usurping and destruction of animal habitat by supplying residential space with tacked-on wildlife corridors and sanctuary containments, or prohibitions on domestic animals, all the while selling altered (humanized) animal habitat at inflated prices because of an imagined and romanticized ‘ethical’ interaction between people and animals. These developments have further distanced animals from humanized place under cover of a cloak of ‘green’ objectification. Zonings that spatially separate humans and animals, and planning codes that specify optimum human-quality habitat, perpetuate this divide, privileging human habitation over nature under the guise of environmental best practice [8].

It has been argued that humans are potentially more committed conservationists when they have opportunities to engage in long-term, everyday interactions with nature [10]. Positive interactions are said to assist humans to construct intangible meanings and attachments in their spaces and places of encounter with nature, fostering connectivity. Likewise, negative interactions and associations (e.g., cruelty to animals, hunting and trapping) are argued to co-occur with domestic and other violence and crime in adults and children [11,12,13].

Despite the social capital, identity, and caring and ethical networks that animals help to form [7,14] these intangible values remain subordinate to the powerful tentacles of industrial capital and institutional instrumentalism as they co-opt nature for their own purposes. In the final section of this paper we show how an ecoversity approach to education might be able to re-situate engaged learning which not only puts into place practical interactions between humans and animals, but also stimulates and harnesses the intellectual and scholarly resources necessary for students and engaged communities to be able to interrogate and change their values, beliefs and behaviors as well as the traditional philosophical and ethical formulations which enable animal objectification.

The assumed division between domestic and wild animals is questioned by Wolch [7], who suggests that any natural boundary is permeable. Humans have domesticated some animals through an imagined but convenient ‘separation’ from wildness, while valuing wild animals as utilities and commodities to be consumed in wildlife reserves and zoos, as a resource to be mined and ‘managed’ for short-term economic gain and entertainment, or blamed for the consequences of poor farming practices.

Such entrenched political and economic forces and self interest need to be countered by more ethical ways of thinking about and relating with non-humans [15]. Smith [4] critiques commercial interests as systematizing ‘distantiation’, that is, the physical and moral separation of supposedly objective authorities from the effects of their regulations and from the place-based values and activities of the (human and non-human) residents they regulate. He asserts that our laws perpetuate the social order with a scientistic perspective that cannot and does not account for the power relations that oppose human-nature equity and a genuine dialectic.

## 3. Ethical Space and Place

An alternative way of conceptualizing animal/human relations and thus addressing animal cruelty and objectification can be found in Wolch's [7] application of a trans-species framework to urban space. Urban areas are the most distanced from animals, particularly wildlife, and her framework argues that they can be re-naturalized through the creation of human-nature proximities, networks of kinship, care and solidarity and situated knowledge that steer dominant structures towards animal-centered standpoints. The trans-species framework theorizes space as a ‘temporal polyvocality’, a space that at different points in time requires different understandings of human animal standpoints and different negotiations of animal and human interests. The approach does not exclude other animal beings, nor does it relativize difference to make people inert to dominant structures. Rather it harnesses the similarities and diversities of human nature and being to create a ‘zoopolis’, in which shared spaces of nature and culture, human and animals are ordinary and everyday. It is also a place where non-humans conspicuously affect humans to such an extent that it becomes necessary to transform oppressive structures that threaten their existence or destroy habitat and environment. In this paper we propose the ‘ecoversity’ to connote these characteristics within an ethical engaged learning framework that embraces the theoretical and practical.

Smith [4] likewise proposes such a situated ethical framework. His geography of space and place installs ‘natural’ laws as the governing societal instruments, and his ‘ethics of place’ connect morality with physical space, helping to create Wolch's [8] ‘moral landscape’ of re-animated urbanism. In Smith's [4] ethical space, ‘context’ is important and requires that humans know non-humans from a closely engaged perspective, as well as from a respectful ‘distance’, as with any interaction of mutuality. Such a sensitivity and sensibility is not fooled by institutional (mis)representation of the non-human world, but stems from an ethical space in which actions and relations are known by all actors, in which people and institutions are open and sensitive to change, and where nature is active in framing the responses of governing institutions.

An ethics of space is territorially and place specific, building relational ways of being between humans and non-humans in each unique context and locale. Buller and Cesar [16] connect ‘discourses of quality and animal welfare… [with] … notions of rurality, territorial specificity and environmental sustainability’. In other words, residents who identify with a place become horrified at the commodification of ‘their’ animals on an industrial scale. They prefer to consume (in its broadest sense) the non-human world in more ideal terms; for example to prefer animal products associated with representations of human-animal rurality as a romanticized, nostalgic and longed-for place and to equate animal welfare with a sustainable future.

These idealized notions about ways of being that are territorially specific, help create the ethical place-based, more-than-human worlds to which we aspire. These notions are similar to Cronon's [17] notion of ‘home’. Cronon [17] has long advocated that the natural and social dualisms be abandoned, suggesting instead that there is a common middle ground in the natural and social landscape that is our ‘home’, a place that is rich with encounter. He epitomizes home as a place of belonging, where cruelty and objectification of our family members could never reside. Home: ‘after all, is the place where finally we make our living. It is the place for which we take responsibility, the place we try to sustain so we can pass on what is best in it (and in ourselves) to our children’ [17].

Other geographers, too, suggest that place-based ethics can generate positive encounters. For example, Johnston [18] urged that Ingold's notion of a dwelt geography could allow human non-human relations and lifeworlds to be understood through different disciplinary lenses and ways of being, with broader scholarly approaches. As such, there is need for an education that offers an ethical space and place to learn and understand ‘the practice and experience of co-relationality’ [18]. Orr [5] includes a dialogue with place as one of his six foundation requirements for enabling people to practice what they learn about living sustainably: ‘…for inhabitants, education occurs in part as a dialogue with place and has the characteristics of good conversation…But true conversation can occur only if we acknowledge the existence and interests of the other’ [5].

Humanist Zygmund Bauman [19] relates matters of learning to the identification of two types of communities of place: the aesthetic and the ethical. The aesthetic community is characterized by perfunctory and ephemeral attractions and bonds between homogeneous participants, designed for immediate gratification and where difference and disorganization are not valued (‘mixophobia’). ‘This kind of communal unity rests on segregation, division, and keeping of distance’ [19]. For Bauman [20], the ideal form of togetherness in a community, ‘being-for’ as opposed to ‘being-alongside’ or ‘being-with’, occurs in the ethical community where communal unity rests on difference (‘mixophilia’), long-term commitment, sharing, concern and responsibility [19].

How we move beyond the concept of an ethical space in which the divide between wild animals and humans might be overcome connotes how humans perceive themselves and the ‘other’ in the presence of non-human animals and the processes of learning. The first of these is considered in the next section.

## 4. Seeing Ourselves Differently

In the text *The animal that therefore I am (more to follow)* the French philosopher Jacques Derrida [2,3] deconstructs the Cartesian idea that humans are thinking animals. If we are to regard animals, like ourselves, as thinking, what else might follow from such a proposition? Derrida's exposition enables us to explore the logical correlates of the proposition, the bizarre leaps of illogic that serve as the basis of our current anthropocentric ways of perceiving and understanding ourselves and animals, and the cruelty to animals that results.

The implications of Derrida's [2,3] arguments are not simply that we need to supplement our current ethical formulations with more inclusive approaches, better able to take into account animal lives and spaces. At the same time we need to *learn how to see ourselves in the presence of non-human animals*, and acknowledge the ways in which our forms and modes of thinking have led to our current condition of blind brutality towards animals. Like Plumwood [21], Derrida [2,3] questions the premises of animal ethics which emphasize similarity, for these require humans to serve as the prototypes of some form of meritorious or superior being worthy of living, while disregarding the alterity of animals; a difference and otherness that exists in their own terms and possibly in a form beyond our comprehension.

For Derrida it follows that:

- we may be entirely unable to comprehend how animals perceive us even as we might recognize their efforts to communicate and our responsibility to respect and protect them. That animals also ‘suffer’ is the real requirement of relationism with humans, not whether there are physical differences;
- at the same time as recognizing ourselves as animals, in distinguishing ourselves as special and ‘thinking’ we manage to conceal the actual diversity of all living beings, including the human animal. Indeed the use of the term ‘animal’, or indeed ‘wildlife’ in relation to ‘human’ indicates an effort to elide the astonishing diversity of ‘animals’ so as to amplify our own uniqueness and difference from the homogeneous ‘other’;
- we have no means of expressing what humans are—only what we are not. Western philosophy is unable to advance understanding of the beings that we actually are. It is only by regarding animals as bestial, cruel and inhumane that we are able to locate ourselves as human, cultured, civilized and superior;
- we have little comprehension of the historical, philosophical, rhetorical and religious forms of thought that limit the kinds of questions we can ask about ourselves and about animal others; questions such as does their ability to communicate mean they talk? Does their similarity to us mean they are like us? Are we responsible for beings we eat and upon which we experiment? These questions are formulated through cultures and particular forms of philosophy and ways of thought and expression; and
- the conceptual and actual violence humanity perpetrates against the ‘animal’ may constitute our very notions of responsibility and ethics. In other words, linguistic and physical violence against animals may in fact form the foundations of our capacity to think and conceptualize responsibility and ethics. For Derrida, an inability of humans to take responsibility for the living (in whatever animal capacity that might reside) requires a different ethics that cannot be found in liberal justice and rights arguments or utilitarian calculation [22].

In Derrida's [2,3] view western philosophy limits our ability to understand ourselves—our being. We are only able to understand ourselves in relation to animal beings. In fact, in our human way of being, animal existence is only relevant when and because it relates to humans. Moreover, our representations of animals enable humans to be defined or understood as civilized, cultured, and superior because animals are uncivilized, uncultured, inferior and bestial. In other words, our understanding of animals validates an understanding of ourselves that occludes understanding of animal-beings in their own terms.

We ask the same question as Cora Diamond [23] and wonder whether the human mind does have the capacity to comprehend an encounter with an animal beyond its object. Can the human mind comprehend the extreme brutality to the animal as Coetzee's [24] Elisabeth Costello did, or do we find difficult or painful things resistant to our thinking, why and how can this be changed? Is an ethical transformation through an encounter of the Derridian kind with an animal, particularly a wild animal, really possible in humans to the extent that it turns around the burgeoning trend in cruelty to animals and to the kangaroo in particular?

Like ecological feminism [21,25], Derrida's work offers an important critique of the problem that dualisms and binary forms of thought create for an ethics which addresses humans and animals [24]. These critiques suggest that we need to go beyond the provision of optimal material conditions for engaged learning to facilitate the development of relational ethics. If we do not want to end up with the same modes of thought and expression that have led to contemporary forms of barbarity, learning engagements also need to enable challenges to established modes of thought and expression about ourselves and animals [26].

## 5. Education, the ‘Ecoversity’ and the Relational Ethics of Place

Levinas [27] has defined a relational ethic as an ‘ethics of encounter’, where the ‘other’ is revealed through its difference from us. Derrida [2,3] however notes that a relational ethic can only manifest if, having become aware of an engagement need with a different ‘other’, there is responsibility for acquiring new knowledge and taking action beyond the encounter, as it opens up possibilities for a future that no prior knowledge might have identified. To suggest a human/animal or human/nature binary or separation is to assume a reductionist or universalist approach that only strengthens any such divide and limits our learning about future directions.

Engaging with difference assumes no pre-determined conclusions about the particular ‘other’, or the direction the engagement might take. There are no relevant principles or rules governing the acquisition of knowledge from the other through the engagement process, although as Buber [28] explains a prelude to this requires a resistance to objectification and an acceptance of mutuality in understanding.

Taking up Derrida's challenge that a relational ethic requires a responsibility for action beyond mere encounter, to enable new possibilities for a future that otherwise-acquired knowledge would not provide, leads us to the proposition of the ‘ecoversity’ as a theoretical and practical construct, which we discuss in this section.

Universities, like urban living spaces, are often imagined as devoid of any connectedness to nature. Like urban living spaces, universities are places where we interact with other humans and where we dwell for a time but generally do not better understand our responsibility for the suffering or wonder of the other. Some, [5,29], have argued that universities are no more than production lines for student qualification, research publication and citation production, and prestige and revenue-seeking entities.

A great deal of what passes as knowledge is little more than abstraction piled on top of abstraction, disconnected from tangible experience, real problems and the places where we live and work [5].

For David Orr [5], it is not simply global governance that has failed us, but also the failure of education to educate. Education has offered few clear directions and conceptual tools to assist us to better understand ourselves and our responsibility to lead the world towards a more sustainable future. Indeed, many of the environmental problems we now face were actually created by educated people and this suggests that we need a different education, not more of the same [5].

Elsewhere, we have concluded that universities have failed Boyer's [30] test of the common good.

There is an assumption that the engaged relations between a university and its regional and local community are about creating something that is good for society and the environment in the traditional Dewey [31] and Boyer [30] way. In a heavily dominated neo-liberal world this public good perspective is a hopeful generalization as, despite well-publicized individual engagement good news stories, we know (however unfortunate or unfair it sounds) that many universities engage only consequentially for recognition, prestige and power [32].

Garlick *et al.* [26] argue that universities ought to function primarily as a public good and thus have an ethical responsibility toward redressing the human-nature divide. They propose that universities adopt a relational ethics and transform themselves as spaces and places wherein their residents might live a ‘mutually engaged’ existence with wildlife and local ecosystems. This vision also taps into the community engagement role that universities are increasingly required to adopt, connecting their teaching and research core business activities with the place in which they stand. Community engagement, by definition, suggests mutuality and reciprocity both within and external to the university. A relational ethics of place results from a genuine relational engagement that brings mutual benefits and addresses conflicts, for example over the use of resources, in terms which take account of all standpoints and interests to build deeper and broader networks and relationships [14].

Universities can thus play a number of roles in creating a relational ethics of place. They provide the space and location for the mutual existence of human and non-human communities. Within that space the engagement with the non-human community is exemplified and fostered through ordinary and everyday proximities and encounters of humans and, more formally, through teaching and research activities. These activities occur within and across disciplines (physical and metaphysical) to address animal geographies, wildlife ecologies, social ethnographies, ethology and ethics.

The concept of the ‘ecoversity’ offers universities and communities an innovative framework through which to undertake sustainability transformations in directions that are underpinned by a relational ethics of place. It is an approach that enables universities to engage the human capital of their communities with that of local human and animal communities to address local and global sustainability matters in practical ways. Through relational place-based engagements in learning, knowledge production and distribution in particular locations, the ‘ecoversity’ can be a vehicle for the practical implementation and further development of an emerging theory of ethics. This form of relational ethics sees learning and research connected to community engagement and global concerns [30] in teaching, research, governance, greening operations and wildlife matters [31].

The ‘ecoversity’ concept has at its heart the notion of leading by example to ensure that daily activities engage students and communities in understanding and active participation in what it means to address the ‘unsustainable core characteristics of our time’ [33]. It provides a framework for engaged learning and transformation and so connects but goes beyond ‘green campus’ and ‘sustainable curricula’ developments into multivalent engagements though university communities, and with the university and other communities [34]. It is a framework for all universities to engage on sustainability matters with local and global communities. The ecoversity notion is thus fundamentally a holistic approach to education for sustainability based on ecological values and ethics. It is an approach which models practical and local applications of those principles in engagements through:

- Campus operations, estate and buildings, wildlife, energy, water, recycling (green campus);
- Curriculum and pedagogy (ecoliteracy and sustainability literacy);
- Research, innovation, policy and planning for the common good; and
- Engagement with community, businesses, schools and local and international partners.

**Figure 1:**
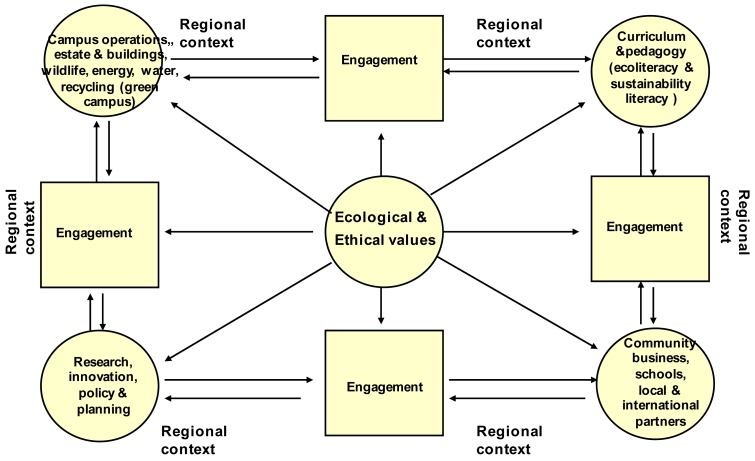
Ecoversity schematic model.

Figure 1 is a refinement of a previously developed schematic [24] which locates ecological and ethical values at the core of the ecoversity approach to take into account both natural and artificial systems.

Examples of university efforts at engaging with sustainability beyond the ‘green campus’ can be found in the University of Plymouth and the University of Bradford in the U.K. However in overlooking the significance of animals and wildlife, these initiatives miss the opportunity to provide a more radical and ethical accounting for sustainability in relation to ecological and ethical values. The University of Bradford branded itself an ‘ecoversity’ in 2005. Initially concerned with the greening of campus estates, Bradford took the opportunity to explore ways of promoting the health and wellbeing of staff and students, to create stronger links with local communities and to undertake design and construction work based on agreed sustainability criteria. Ecoversity was established as a university program with a program manager and board to oversee the development of four project objectives: environment, community, education for sustainable development and economy. The University of Plymouth was awarded a Centre for Excellence in Teaching and Learning—Education for Sustainable Development (CETL-ESD) award from the Higher Education Funding Council for England for a five-year period from 2005. The award was in recognition of existing and potential excellence in the sustainability field and provided for the establishment of a Centre for Sustainable Futures (CSF), with a remit to transform the University into ‘an ‘institution modeling university-wide excellence in sustainability’. To accomplish this aim CSF developed the ‘4C’ approach to change, addressing the four dimensions of curriculum, campus, community and (institutional) culture. The model is the basis of the university's sustainability policy and strategic action plan to ensure the embedding of sustainability beyond 2010.

Following Sacks [35], the goal of the ecoversity is to teach us what we are a part of. It does this by sharing knowledge, identifying local/global problems and solutions, stimulating ethical debates and challenging unsustainable development and the excesses of transnational capitalism [34]. It is not therefore that sustainability should be integrated into universities, but rather that universities need to transform themselves into the integrated holistic communities implied by sustainability perspectives [36].

As places of knowledge and learning we see a public good responsibility for universities to address the burgeoning problematic of the human-animal divide and the cruelty and brutality it connotes through a relational ethic beyond mere encounter. The engaged learning framework of the ecoversity is a mechanism for this to be achieved and we invite universities to take up this common good challenge in meeting the Boyer test of a worthwhile university in a modern day context.

## 6. Lessons in the ‘Ecoversity’ for Conservation and Welfare

According to a 2008 WWF report *2010 and Beyond: Rising to the Biodiversity Challenge* [37], ‘Australia already has the worst rate of mammal extinctions in the world’ and ‘40 per cent of mammal extinctions globally in the last 200 years have occurred in Australia’. Despite a relatively small population, Australians have managed to wreak havoc on a unique and fragile natural environment in a very short time period.

Governments in Australia and worldwide appear unable to act effectively on these critical environmental issues—a consequence perhaps of decades of entrenched post-colonialism, managerialism, cerebral capitalism and neoliberal practices. Through the internet and global media, a dual local-global phenomenon is now appearing. Environmental activism, previously predominantly related to local place-based environmental issues, has taken on a global relevance, and conversely, rapid engagement with and implementation of global environmental agendas has underlined, and renewed awareness of their significance at the local level [38].

In their accounts of institutions and processes designed to address global environmental governance Martello and Jasanoff [38] argue that three things need to happen. First, global environmental governance solutions require local opportunities for expression; second, we need to realize that the identification, understanding and representation of environmental problems relates to the ways in which we choose to address problems. In other words, environmental knowledge is not objective or distinct from the power-knowledge formations of science and the local and national and supra-national politics that identify certain problems as meriting attention. Finally, effective governance requires innovations in power-knowledge formations to achieve well-articulated mechanisms of communication, translation and interaction. We believe that the ‘ecoversity’ fulfills these criteria for environmental action.

Whenever conversation turns to wildlife the discussion is invariably about science-based conservation, with vague quantitative terms such as ‘abundant’, ‘threatened’, ‘endangered’ *etc.*, guiding anthropocentric behavior in justifying captive breeding, ‘management’, eradication, habitat change or similar institutional intervention programs. There is no discussion of cruelty and brutality and the animals in question are simply objects to be counted. If it is calculated (generally with considerable imprecision) that there are ‘too many’ of a given species for a given situation, the conservationist seeks resources to reduce their numbers for the benefit of a wider ‘ecology’. When it is calculated there are ‘too few’ animals in a given situation to remain independently viable in the wild, resources are sought to increase their numbers through various institutional containment and breeding programs. These approaches to both situations are a product of the difference between the methods and values of deep and shallow ecology, as well as the values of the institutional authority. Unfortunately for the welfare of these animals these institutional values are generally based on neoliberal principles of economic rationalism, anthropocentrism and instrumentalism. The arena is restricted to science and institutionalism and other valuable knowledge from a range of appropriate disciplines and experiences critically relevant to the future of animals is excluded.

It might be argued that anthropocentric quantification is also a characteristic of the utilitarian sentience [39,40] and ‘rights’ respect-based [41] approaches to reducing animal cruelty. As sentient beings, non-human animals ought to have ‘rights’ to pursue the ‘fundamental interests’ appropriate for their species, as humans would expect to have rights to pursue their basic living requirements. According to Cavell [42], the ethical outcome in the Singer approach is to ‘tally up the ‘interests’ of the particular beings in question in a given situation, regardless of their species, and would determine what counts as a just act by calculating which action maximizes the greatest good for the greatest number…’.

Derrida [2,3] would agree with the objectives of Singer and Regan aimed at reducing animal cruelty, because it will: ‘…awaken us to our responsibilities and obligations with respect to the living in general, and precisely to this fundamental compassion that, were we to take it seriously, would have to change even the very basis…of the philosophical problematic of the animal’ [2].

However, the means of achieving this ‘awakening to our responsibilities’ are different for Derrida. For Derrida, as we have seen, the way to reducing animal cruelty is through the transformative experience and knowledge acquisition that occurs in an awareness raising encounter (contextual ethics) between human and non-human animals of the kind experienced by Coetzee's [24] Elizabeth Costello in *The Lives of Animals*.

We believe the ‘ecoversity’, with its foundation in place-based relational ethics, provides more than a mechanism to help bridge the gap between human and non-human animals and that a transformative encounter can generate the knowledge to foster creative and ethical solutions to wildlife welfare and issues of cruelty. It therefore has the potential to assist to resolve the conservation and animal welfare dichotomy, as well as to open pathways between science and understanding. The ‘ecoversity’ model of involvement and knowledge acquisition through encounter enables such a dialogue because it does not promote quantitative anthropocentrism, but rather understanding through relationism.

We also believe the concept of the ‘ecoversity’ addresses the real concern where humans’ growing physical divide from wildlife (and animals generally) in modern society has reduced any possibility of an encounter with the eyes of a wild animal. The question about Derrida's ‘surprise’ encounter with the eyes of his famous household cat not being representative of a genuine encounter with otherness can be addressed through the ‘ecoversity’ as the notion of ‘surprise’ or the ‘rawness of nerves’ manifested by Coetzee's Elisabeth Costello. Those of us with daily and close involvement with wildlife never cease to have the ‘surprise’ encounters that awaken us to the possibilities of new knowledge to address the human/ non-human animal divide. The framework of the ‘ecoversity’ formalizes, widens and connects the learning process.

## 7. Conclusions

In this paper we have been concerned to bring new thinking to reducing burgeoning wildlife cruelty in a neoliberal world by actioning processes of engaged learning within an open spatial context using the Derridian relational ethic associated with a transformative encounter with otherness, *i.e.*, wildlife. We have proposed the model of the ‘ecoversity’ as providing the engaged learning framework that can connote these perspectives to enable real on-the-ground change to occur to alleviate cruelty, in contradistinction to the animal cruelty impact of the dominant anthropocentric institutional instrumental paradigm that continues to be entrenched in modern day society.
